# The benefit of early‐stage diagnosis: A registry‐based survey evaluating the quality of life in patients with melanoma

**DOI:** 10.1002/ski2.237

**Published:** 2023-04-17

**Authors:** Jade N. Young, Kelly Griffith‐Bauer, Emma Hill, Emile Latour, Ravikant Samatham, Sancy Leachman

**Affiliations:** ^1^ Department of Dermatology Oregon Health and Science University Portland Oregon USA; ^2^ The Polyclinic Seattle Washington USA; ^3^ Biostatistics Shared Resource Knight Cancer Institute Oregon Health and Science University Portland Oregon USA

## Abstract

**Background:**

The morbidity associated with advanced stage melanoma is an important consideration in the dialog surrounding early detection and overdiagnosis. Few studies have stratified melanoma patient quality of life (QoL) by stage at diagnosis.

**Objective:**

We sought to investigate if melanoma stage is independently associated with changes in QoL within a large, community‐based melanoma registry. Secondarily, we investigated whether demographic factors such as age, geographic location or level of education are associated with changes in QoL in the same population.

**Methods:**

1108 melanoma patients were surveyed over a three‐month period using the QoL in Adult Cancer Survivors Survey, consisting of 47 items on a 7‐point frequency scale. Data were analysed using both descriptive statistical models and adjusted multivariate logistic regression.

**Results:**

There were 677 respondents generating a 61% response rate. Overall, higher stage at diagnosis correlated with the largest decreases in QoL as it pertained to both general (*p* = 0.001) and Cancer‐Specific stressors (*p* < 0.001). Education level (*p* = 0.020), age (*p* < 0.001), rural area code designation (*p* = 0.020) and family history of melanoma (*p* = 0.017) were also independently associated with changes in QoL.

**Conclusion:**

Earlier stage at melanoma diagnosis is associated with better QoL and thus represents a crucial intervention in patient care. Given our findings and the growing body of evidence surrounding morbidity in late‐stage melanoma, it is essential that QoL be included in assessing the benefits of early detection.

## Graphical abstract

The morbidity associated with advanced stage melanoma is an important consideration in the dialog surrounding early detection and overdiagnosis. Few studies have stratified melanoma patient quality of life by stage at diagnosis. We sought to investigate if melanoma stage is independently associated with changes in quality of life within a large, community‐based melanoma registry. We found that melanoma diagnosed at a later stage is associated with reduced quality of life as measured by distress over recurrence, finances, appearance, family, cognitive problems, fatigue, pain, sexual problems, and social avoidance. Thus, quality of life is an essential consideration in assessments of melanoma screening programs and early detection efforts.

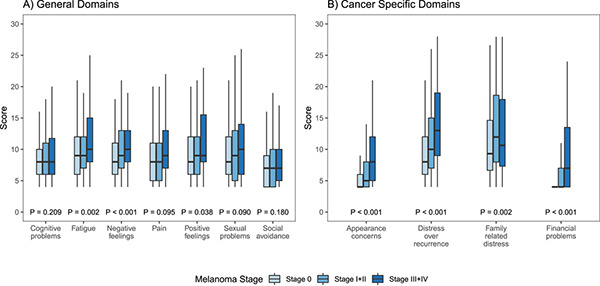

**What is already known about this topic?**

- Heightened screening increases the detection of thin melanomas. This practice promotes concern that thin melanomas are overdiagnosed and would not result in fatal disease if left untreated. Conversely, patients and physicians alike contest that mortality is not the only important metric and that quality of life (QoL) should be considered as well. Few studies assessing the benefit of melanoma early detection have considered patient QoL and melanoma‐associated morbidity.

**What does this study add?**

- We found that melanoma diagnosed at a later stage is associated with reduced QoL as measured by distress over recurrence, finances, appearance, family, cognitive problems, fatigue, pain, sexual problems, and social avoidance. Thus, QoL is an essential consideration in assessments of melanoma screening programs and early detection efforts.

## 1 BACKGROUND

Melanoma diagnosed at the localised stage, prior to metastasis, is associated with a very favourable prognosis (5‐year survival rate of 99.5%), whereas regional and distant metastasis is associated with 5‐year survival rates of only 70.6% and 31.9%, respectively.^1^ Most patients and physicians prioritise mortality in shared decision making conversations, however many patients feel quality of life (QoL), including morbidity, is also a crucial consideration.^2^ With the introduction of several novel targeted and immunotherapeutic agents, long term survival of patients with melanoma has become increasingly realistic and QoL during survivorship must be prioritised.^3^ QoL has been recognized by the Food and Drug Administration as an important metric in the approval of new therapies.^4^, ^5^ Because of this, recent therapeutic trials in melanoma research, including pembrolizumab and v‐raf murine sarcoma viral oncogene homolog B1 (BRAF) inhibitors, have begun to incorporate QoL measurements in their protocols.^6^, ^7^, ^8^ Despite the emphasis on QoL in the drug approval process, there has been little data available to stratify QoL by stage at diagnosis and the topic has been minimally incorporated into the conversation surrounding early detection programs.

The impact of melanoma screening and early detection is an area of current controversy. In 2016, the United States Preventative Service Task Force issued a statement that there is insufficient evidence to support recommending preventative skin cancer screenings in asymptomatic adults.^9^ There have been concerns raised regarding the overdiagnosis of melanoma, defined as the detection of cancer that would not result in clinically significant disease without treatment.^10^, ^11^, ^12^ These concerns are born out of similar dialogs in lung, prostate, breast, and thyroid cancer.^13^ Concerns are grounded in the failure of studies to show that screening programs reduce rates of melanoma, discordance in the histopathologic and dermoscopic diagnosis of thin melanomas,^14^, ^15^, ^16^ and the suggestion that melanoma early detection does not improve outcomes. Authors cite data showing that the incidence of thin melanoma has increased while melanoma related mortality has remained stable or slightly decreased. This has been referred to as the “high prevalence, rare death mismatch.”^10^, ^17^, ^18^, ^19^

Conversely, recent evidence demonstrates that rates of thin melanomas have actually stabilised in the last decade whereas thick melanoma incidence has increased with a disproportionate impact on minority groups and those of low socioeconomic status.^20^ Those in support of heightened screening efforts argue the risk of tumour progression poses greater concern than overdiagnosis.^21^, ^22^, ^23^ Although, on average, thin melanomas are associated with better outcomes, there is currently no marker for lethality of melanoma, so risk for metastasis and death exist for any melanoma. Melanoma can be an aggressive malignancy, and current diagnostic methods cannot perfectly predict which tumours will become metastatic.^24^ In fact, an estimated 25% of melanoma related deaths are due to thin melanomas.^25^ Personal history of melanoma in situ is also a risk factor for developing future melanomas and other cancers.^26^, ^27^ Because heightened screening increases detection of thin melanomas,^17^, ^28^, ^29^ investigation is needed to determine how earlier stage at diagnosis affects all aspects of patient outcome, including patient morbidity and QoL.

Studies evaluating the impact of screening programs have not considered the impact of delayed diagnosis on QoL.^18^, ^19^ Only a few small studies have compared Cancer‐Specific QoL measures between patients with early and late stage melanoma.^30^, ^31^ Therefore, we utilised the QoL in Adult Cancer Survivors Survey (QLACS)—which has been validated in patients with cancer,^31^ to objectively evaluate the QoL of melanoma survivors and determine if stage of melanoma at diagnosis is independently associated with changes in QoL. Secondarily, we investigated whether demographic factors such as age, geographic location or level of education are associated with changes in QoL in the same population. With this investigation, we hope to elucidate factors that influence the care that dermatologists provide and highlight possible opportunities to improve patient QoL.

## 2 METHODS

### 2.1 Study design and participant selection

This study was a cross‐sectional assessment of QoL in melanoma survivors in all stages of disease (0‐IV) using the QLACS, a validated QoL tool designed to assess issues relevant to long term cancer survivors.^32^ This instrument was carefully selected among an armamentarium of QoL surveys because of its specific focus on financial toxicity, emotional well‐being, physical health, pain, and appearance‐related concerns. A total of 1108 participants with a self‐reported diagnosis of melanoma from an IRB‐approved Melanoma Community Registry^33^ (OHSU IRB#10561) were invited to complete the QLACS between August and December 2015. Demographic information and melanoma‐specific data (age at time of diagnosis, stage at time of diagnosis, family history of melanoma, melanoma location, number of locations involved, and years since diagnosis) specifically associated with each participant was completed by participants during registry enrolment. Descriptive language was utilised within the survey to guide participants to select the correct stage of disease. For example, for stage IV, the answer choice was as follows: “I believe melanoma spread to other parts of my body because signs of melanoma have been seen by imaging studies like computed tomography, positron emission tomorgraphy, or magnetic resonance imaging. You may have been told this was Stage IV disease and may have received chemotherapy, targeted (e.g. BRAF) therapy, or immunotherapy (e.g. Ipilimumab or Nivolumab).” We received 677 responses (61% response rate). Study data were collected and managed using REDCap electronic data capture software.

### 2.2 QLACS survey:

The QLACS is comprised of 47 items with a 7‐point frequency of experience scale, ranging from 1 (never) to 7 (always) with respect to the last 4. Higher scores correlate with reduced QoL. Twelve categories of questions are grouped into Cancer‐Specific and General Domains. The combined domain is the sum of the two domains. The Cancer‐Specific domain includes questions regarding distress related to appearance, recurrence, family, or financial problems. The General Domain includes cognitive problems, fatigue, negative feelings, pain, positive feelings (reverse scored), sexual problems, and social avoidance. Based on each participant's responses in each of these categories, a summary score was calculated for general and Cancer‐Specific domains based on the previously validated scoring protocol.^32^ In prior cancer studies, patients with breast and ovarian cancer tend to have total combined QLACS summary scores greater than 100, indicating reduced QoL, compared to healthy controls.^32^, ^34^ For example, the average QLACS combined score in a healthy population is 97.8 and is 129.7 in an ovarian cancer population, suggesting that even small absolute changes in QLACS score can represent entirely different life circumstance.^35^

### 2.3 Statistical analysis of demographic data

Descriptive statistics were used to summarise the demographic characteristics of the survey respondents. Melanoma locations were grouped based on level of visibility to others. Respondent zip codes were classified as “Urban core” (>50 000 people), “Suburban” (metropolitan areas with high commuting flow), “Large rural” (10 000–49 999) and “Small town/rural” (<10 000) using rural‐urban commuting area (RUCA) designation to assess the influence of rural and urban lifestyles on QoL.^36^

### 2.4 Statistical analysis of general and Cancer‐Specific Domains

Two separate linear regression models were fit: one using the General Domains score as the outcome, and the other using the Cancer‐Specific Domains score as the outcome. Candidate variables were screened for univariable associations with the outcomes using a threshold *p*‐value <0.25. Final models consisted of screened variables with *p*‐values <0.05 in a multivariable model. Surveillance, Epidemiology, and End Results reports stage I and II as a combined group (I/II), and the same approach was used in our analysis. Stage III and IV were grouped due to low sample size of stage IV participants. Due to the high percent of missingness (Table S1, Table S2), multiple imputation was employed as a sensitivity analysis where many plausible complete data sets were created and analysed for both General (Table S3) and Cancer‐Specific domains (Table S4). Estimates from complete case models and the multiple imputation models proved to be similar, thus complete cases (i.e. subjects without any missing outcome or model variables) were used in this analysis. Analyses were performed using R: A Language and Environment for Statistical Computing.^37^

## 3 RESULTS

### 3.1 Participant demographics and cancer characteristics

Of these 677 participants, all of whom are self‐reported patients with melanoma, 425 (62.8%) identified as female and 219 (32.3%) identified as male. Most respondents—626 (92.5%)— self‐reported as white, and 631 (93.2%) were neither Hispanic nor Latino. There were 515 (76.1%) participants living in an urban area code, while 71 (10.5%) lived in a “large town,” 58 (8.6%) lived in a suburban area code, and 22 (3.2%) lived in either a small town or rural area. On average, participants were 8.76 (SD 7.60) years past their date of melanoma diagnosis. Regarding family history, 210 respondents (31.0%) had a family history of melanoma, while the remaining respondents either did not know or reported not having a family history of melanoma. Regarding the highest level of education, those participants having completed either some college, a 4‐year college degree, and post‐graduate or professional degree represented 10.2%, 20.4%, and 27.2%, respectively. Those completing high‐school or equivalent degree represented 9.6% of the sample. Most participants had either Stage 0 (299, 44.2%) or invasive localised (Stage I or II, 330, 48.7%) melanoma. Regarding stage, 47 participants (6.9%) represented advanced‐stage disease. Roughly half (50.4%) of participants had single‐location melanoma, while 18.2% had two or more locations. In terms of visibility, 349 (51.6%) stated their melanoma “could be hidden if bothersome” while the remaining participants either reported having visible melanomas or did not answer (Table 1).

**TABLE 1:** Demographic information of respondents.

Category	Mean
*N*	677
Age (mean (SD))	57.91 (13.06)
Age (%)	
20 to 29	12 (1.8)
30 to 39	54 (8.0)
40 to 49	86 (12.7)
50 to 59	134 (19.8)
60 to 69	180 (26.6)
70 or older	111 (16.4)
Missing	100 (14.8)
Gender (%)	
Male	219 (32.3)
Female	425 (62.8)
Prefer not to answer	0 (0.0)
(Missing)	33 (4.9)9
Race (%)	
Native American/Alaskan native	0 (0.0)
Asian	0 (0.0)
Black or African American	1 (0.1)
Multiple races	10 (1.5)
Native Hawaiian/Pacific Islander	0 (0.0)
White	626 (92.5)
Other	3 (0.4)
Preferred not to answer	4 (0.6)
(Missing)	33 (4.9)
Ethnicity (%)	
Hispanic or Latino	8 (1.2)
Not Hispanic or Latino	631 (93.2)
Prefer not to answer	5 (0.7)
(Missing)	33 (4.9)
Highest level of education (%)	
4‐year college degree	138 (20.4)
High school, equivalent, vocational training, 2‐year degree	65 (9.6)
Some college but no degree	69 (10.2)
Post‐graduate or professional degree	184 (27.2)
(Missing)	221 (32.6)
Urban/Rural (%)	
Urban	514 (75.9)
Rural	134 (19.8)
(Missing)	29 (4.3)
RUCA designation (%)	
Urban core	515 (76.1)
Suburban	58 (8.6)
Large town	71 (10.5)
Small town/rural areas	22 (3.2)
(Missing)	11 (1.6)
Family history of melanoma (%)	
No	314 (46.4)
Yes	210 (31.0)
I dont know	120 (17.7)
(Missing)	33 (4.9)
Personal history other cancer (%)	
No	271 (40.0)
Yes	130 (19.2)
(Missing)	276 (40.8)
Stage (%)	
Stage 0	299 (44.2)
Stage I + II	330 (48.7)
Stage III + IV	47 (6.9)
(Missing)	1 (0.1)
Years since diagnosis (mean (SD))	8.76 (7.60)
Years since diagnosis (categorical) (%)	
Less than 5	195 (28.8)
5 or greater	382 (56.4)
(Missing)	100 (14.8)
Visibility of location (%)	
Can't hide	62 (9.2)
Can hide if bothersome	349 (51.6)
Only visible among close contacts	17 (2.5)
Other	36 (5.3)
Did not reply	213 (31.5)
Number of locations (%)	
1	341 (50.4)
2	86 (12.7)
3	27 (4.0)
4	6 (0.9)
5	4 (0.6)
Did not reply	213 (31.5)

Abbreviation: RUCA, rural‐urban commuting area.

### 3.2 General Domains analysis reveals significant associations between stage, family history, and quality of life

Combined average summary scores for stage 0, I/II, and III/IV were 94, 103.5, and 119.5, respectively. The average summary scores in the General Domain for stage 0, I/II, and III/IV were 62.3, 65.5, and 74, respectively. Having advanced stage or a family history of melanoma were associated with changes in QLACS General Domains scores (*p* = 0.001 and *p* = 0.017, respectively). Adjusting for family history, those with Stage III or IV melanoma scored significantly worse in the General Domain than those with Stage 0 (14.6 points higher [95% confidence interval, CI: 6.7–22.5] *p* < 0.001). Those with Stage I or II scored similarly to Stage 0 participants (3.5 points higher [95% CI: −0.7–7.8] *p* = 0.103). Adjusting for melanoma stage, those with a family history of melanoma had on average, QLACS scores 5.1 points higher [95% CI: 0.9–9.2], indicating lower QoL than those without a family member with melanoma (*p* = 0.017). There were no significant associations between QLACS General Domains scores and age, gender, race, education, RUCA designation, and years since diagnosis (Table 2).

**TABLE 2:** On the left, association of quality of life (QOL) scores and Cancer‐Specific Domains with covariates of interest.

Covariate	Cancer‐specific mean difference in QLACS score (95% CI)	*p* value	General domain mean difference in QLACS (95% CI)	*p* value
Stage		<0.001***^a^ ^,^ ^b^		0.001**
Stage 0	Reference level		Reference level	
Stage I OR II	3.4 (0.5–6.4)	0.022*^c^	3.5 (−0.7–7.8)	0.103
Stage III OR IV	11.6 (6.1–17.2)	<0.001***	14.6 (6.7–22.5)	<0.001***
Age		<0.001***	‐	‐
20 to 29	3.8 (−7.2–14.8)	0.496	‐	‐
30 to 39	2.7 (−2.8–8.2)	0.340	‐	‐
40 to 49	7.0 (2.3–11.7)	0.004**	‐	‐
50 to 59	Reference level		‐	‐
60 to 69	−5.8 (−9.6 to −1.9)	0.003**	‐	‐
70 or older	−8.9 (−13.3 to −4.5)	<0.001***	‐	‐
Highest level of education		0.020*	‐	‐
High school, equivalent, vocational training, 2‐year degree	−0.2 (−4.7–4.3)	0.926	‐	‐
Some college but no degree	0.7 (−3.7–5.2)	0.751	‐	‐
A 4‐year college degree	Reference level		‐	‐
Post‐graduate or professional degree	−4.5 (−7.9 to −1.2)	0.009**	‐	‐
RUCA designation		0.020*	‐	‐
Urban core	Reference level		‐	‐
Suburban	1.1 (−4.3–6.4)	0.691	‐	‐
Large town	4.6 (0.0–9.1)	0.049*	‐	‐
Small town/rural areas	9.2 (2.3–16.2)	0.010**	‐	‐
Number of locations		0.010*	‐	‐
1	Reference level		‐	‐
2	5.5 (1.9–9.1)	0.003**	‐	‐
3 or more	2.6 (−2.6–7.8)	0.334	‐	‐
Family history of melanoma	‐	‐		0.017*
No	‐	‐	Reference level	
Yes	‐	‐	5.1 (0.9–9.2)	0.017*

*Note*: Results for statistically significant covariates using univariable linear regression are shown with adjusted, multivariable results. For reference, in the literature, mean QLACS summary scores in cancer patients within the Cancer‐Specific domain are 38.0 (SD 17.4).^32^ On the right, association of quality of life (QOL) scores and General Domains with covariates of interest. Results for statistically significant covariates using univariable linear regression are shown with adjusted, multivariable results. For reference, in the literature, mean QLACS summary scores in cancer patients within the General Domain are 71.2 (SD 25.6). If there were no significant associations within a group, it is indicated with a dash (−).

Abbreviations: CI, confidence interval; QLACS, QoL in Adult Cancer Survivors Survey; RUCA, rural‐urban commuting area.

^a^ Three asterisks (***) indicates a *p* value between 0 and 0.001, ** indicates it is between 0.001 and 0.01, * indicates it is between 0.01 and 0.05.

^b^ The *p*‐values within the headers indicate statistical significance based on a likelihood ratio for that specific variable, indicating an association without directionality. These values are used in the statistical model building and show why a variable is included in the multivariable model.

^c^ *P*‐values within the broken down groups are based on a Wald test, and compare each level to a reference level with directionality.

### 3.3 Multivariable Cancer‐Specific domains analysis reveals significant effects on quality of life

A multivariable model for QLACS Cancer‐Specific Domains scores was developed to adjust for any potential confounding between factors, and showed significant associations with stage, age, education, RUCA designation, and number of melanoma locations. Average Cancer‐Specific summary scores for stage 0, I/II, and III/IV were 31.7, 38, and 45.5 respectively. Similar to General Domain scores, higher stage was associated with worse QoL scores in Cancer‐Specific domains (*p* < 0.001). Compared to the Stage 0 group, Cancer‐Specific QLACS scores were worse in Stage I/II (3.4 points higher, [95% CI: 0.5–6.4] *p* = 0.02) and demonstrated even greater scores in Stage III/IV (11.6 points higher [95% CI: 6.1–17.2] *p* < 0.001). Older participants tended to score lower within the Cancer‐Specific Domains, indicating better QoL (*p* < 0.001); those with higher education also scored lower (*p* = 0.009). Compared to participants living within an urban core, participants in large towns had higher scores (4.6 points higher [95% CI: 0.0–9.1] *p* = 0.049), and in Small/town rural area scored even higher (9.2 points higher [95% CI: 2.3–16.2 *p* = 0.010) (Table 2).

**FIGURE 1:**
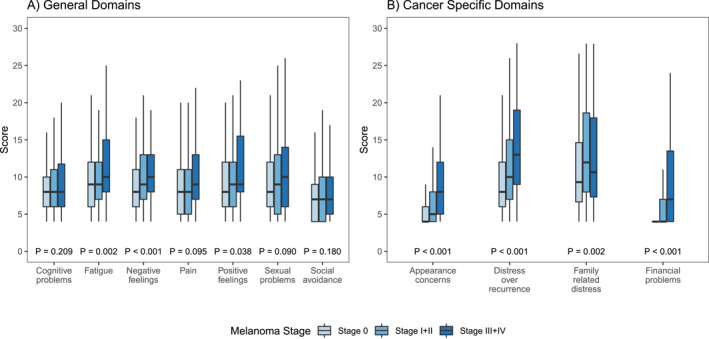
Late‐stage melanoma patients experience decreased quality of life (QoL) in general (a) and Cancer‐Specific (b) domains. *P* values indicate, for each subdomain, if there is a difference in stage group using one‐way analysis of variance.

Since stage showed significance in both multivariable models, we explored distribution of participants' scores across each of the twelve Sub‐Domains while stratifying by Stage (Figures 1a and b). All sub‐domains equally drove the overall difference in QLACS scores as analysed in the Cancer‐Specific analyses (Figure 1a), however fatigue and negative feelings drove the overall differences between stages within the General Domain (*p* = 0.002 and *p* < 0.001, respectively) (Figure 1b).

## 4 DISCUSSION

As melanoma survival rates increase, it is crucial that the dermatologic community quantifies the impact that early detection of melanoma has on morbidity and QoL. The discussion surrounding the value of early detection and screening programs primarily considers mortality rather than morbidity, consists mainly of editorials (rather than data), and is often devoid of the patient experience.^24^, ^28^, ^38^, ^39^, ^40^ Currently available QoL assessments either lack representative sample size, are specific to the effect of systemic therapy, or use surrogate models for QoL assessment rather than information directly from patients.^5^, ^6^, ^7^, ^30^, ^41^, ^42^ Our investigation using a survey based approach of 677 melanoma survivors demonstrates that patients with more advanced melanomas suffer from reduced QoL. Specifically, cancer‐related stress over appearance, family, recurrence, and financial burden represent statistically significant contributors (*p* < 0.01) that worsen QoL with the highest (worst) QLACS scores being associated with the most advanced stage melanomas. There was also a trend towards worse QoL for stage III/IV patients in every domain investigated, with significant associations noted for fatigue, increased negative feelings, decreased positive feelings, and all of the Cancer‐Specific contributors. This indicates that the greatest impacts of late stage detection on QoL are related to emotional, physical and financial state (Figure 1b). Overall, combined summary scores increased as stage increased, indicating worse QoL. Stage 0 disease scores were most similar to that of controls in prior studies (scores less than 100), while stage III/IV disease was most similar to breast and ovarian cancer survivors (scores ranging 100–130).^32^, ^34^, ^35^

Further, lower levels of education are known to be associated with worse outcomes (mortality).^43^, ^44^ Our findings indicate that QoL is also decreased in this population independent of stage of disease or other variables. Those with a graduate degree report better cancer related QoL compared to those with a 4‐year degree. Although no changes were statistically significant between those with 4‐year degrees and a high school equivalent education, the observed trend suggests that increased educational level is associated with better QoL. This may, in part, be a consequence of feeling less prepared to navigate the medical system, or potentially reflect a lack of comfort with supportive materials provided during visits. Overall, it is difficult to ascertain the true significance of these findings and further exploration regarding QoL and education status is warranted.

Younger patients reported decreased Cancer‐Specific QoL as well. The drivers of this are unknown, however potential inferences include the burden of frequent doctor's appointments during years traditionally dedicated to career or family building, cosmetic consequences, and increased overall life‐years of coping with their diagnosis compared to older patients. Family history of melanoma was independently associated with decreased QoL in the General Domain. This may reflect the overall burden of disease on family members, and the trickle‐down effect of familial stress and economic consequences.

The exact drivers for the worsened QoL amongst late‐stage melanoma patients remain incompletely characterised. The utilization of immune checkpoint inhibitors and targeted anti‐tumour therapy in the treatment of advanced stage melanoma is a possible source of worse QoL. Research to date has shown that patients on these long‐term therapies have improved prognosis, but also numerous side effects including autoimmune disease, endocrinopathies, asthma, gastrointestinal issues, dermatitis, nephritis, and hepatitis.^8^, ^45^, ^46^ Because the use of these novel agents are reserved for later‐stage melanoma, it is possible that the side effects of systemic therapy contribute to the overall decreased QoL in late‐stage melanoma.^46^

The COVID‐19 pandemic has been estimated to delay the diagnosis of melanoma by up to 3–6 months and predicted to increase mortality and associated health care costs as a result.^47^, ^48^, ^49^, ^50^, ^51^, ^52^ Given our findings, COVID‐19 related delays in diagnosis are also likely to negatively affect QoL as patients present with thicker tumours. The long‐term impacts of COVID‐19 on dermatologic care may not be known for many years, however the wide adoption of telemedicine during the pandemic may have some benefit to patients with melanoma in rural areas. Our data indicate Cancer‐Specific QoL in melanoma patients varies by RUCA designation, or geographic location. Compared to living in urban core areas, living in a large town was associated with reduced QoL, and further reduction was seen for those living in rural areas. These findings highlight probable issues with rural access to care, which may also contribute to delayed diagnosis and inferior QoL in areas far from large melanoma treatment centres. For example, Morenz and colleagues recently evaluated the barriers to accessing dermatological care in rural Native American communities across the United States.^53^ One of the most pertinent barriers is geographic location and lack of proximity to dermatology clinics. The median driving distance between rural U.S. Indian Health Service hospitals and the nearest dermatology clinic is 68 miles. Overall, 88% of rural counties do not have a dermatologist.^43^ These disparities are glaring for minority and low socio‐economic status communities for which skin cancer mortality rates are disproportionately high.^44^ Targeted solutions may include more robust implementation of teledermatology, as only 9% of rural Indian Health Services in the U.S. report receiving teledermatology services.^53^, ^54^

Surveys are limited by response bias, which can affect the demographic distribution. Recall bias may be an additional limitation. Although stage at diagnosis was self‐reported, survey questions contained language intended to guide participants and minimise recall bias as discussed in the methodology. Time elapsed since diagnosis did not differ across stage groups (*p* = 0.396) suggesting that any recall bias that might exist would be similar across the groups. In addition, subjects were, on average, greater than 8 years out from their diagnosis. This introduces a potential underestimation of QoL within the entire sample. A potential confounder not addressed in our investigation is the use of systemic therapy, a byproduct of the recent approval of several therapies.^55^ Future studies could examine the role of systemic therapy across stage as it relates to QoL.

## 5 CONCLUSION

Our findings suggest that earlier stage at diagnosis, urban living, older age, and higher level of education are all independently associated with improved cancer related QoL in patients with melanoma. Overall, the largest differences in the study were observed when scores were stratified by stage, supporting the conclusion that early detection may be one of the most crucial interventions in adjusting the QoL of melanoma patients. Given our findings and the growing body of evidence surrounding morbidity in late‐stage melanoma, it is essential that QoL be included in assessing the benefits of early detection.

## CONFLICT OF INTEREST STATEMENT

The authors declare no conflicts of interest.

## AUTHOR CONTRIBUTIONS

**Jade N. Young**: Conceptualization (Equal); Data curation (Lead); Formal analysis (Equal); Investigation (Lead); Project administration (Lead); Writing – original draft (Lead); Writing – review & editing (Lead). **Kelly Griffith‐Bauer**: Conceptualization (Lead); Formal analysis (Equal); Investigation (Equal); Methodology (Lead); Project administration (Equal); Supervision (Equal); Writing – original draft (Lead); Writing – review & editing (Equal). **Emma Hill**: Conceptualization (Lead); Data curation (Equal); Methodology (Equal); Project administration (Equal); Writing – original draft (Lead); Writing – review & editing (Equal). **Emile Latour**: Conceptualization (Supporting); Data curation (Lead); Formal analysis (Lead); Investigation (Equal); Methodology (Equal); Resources (Equal); Software (Lead); Validation (Lead); Writing – original draft (Supporting); Writing – review & editing (Supporting). **Ravikant Samatham**: Conceptualization (Supporting); Data curation (Lead); Formal analysis (Supporting); Software (Supporting); Validation (Equal). **Sancy Leachman**: Conceptualization (Lead); Data curation (Lead); Formal analysis (Equal); Funding acquisition (Lead); Investigation (Lead); Methodology (Lead); Project administration (Lead); Supervision (Lead); Writing – original draft (Equal); Writing – review & editing (Equal).

## ETHICS STATEMENT

Reviewed and approved by Oregon Health & Science University IRB, approval #10561.

## Supporting information

Supplementary Material

## Data Availability

The data presented in this manuscript may be made available upon request, at the discretion of the investigator, with investigator support.
