# Supplementary material for: The benefit of early‐stage diagnosis: A registry‐based survey evaluating the quality of life in patients with melanoma
Source: Skin Health Dis. 2023 Apr 17;3(4):e237. doi: 10.1002/ski2.237 (PMC10395626; doi:10.1002/ski2.237)
Supplement: Supplementary file 1 — Supplementary Material [file SKI2-3-e237-s001.docx]

**Supplemental Online Content**

**The benefit of early stage diagnosis: A registry based survey evaluating quality of life in patients with melanoma**

Authors:   Jade N. Young BS^1*^, Kelly Griffith-Bauer MD^1,2*^, Emma Hill MD^1*^, Emile Latour MS^1,3^, Ravikant Samatham PhD^1^, Sancy Leachman MD, PhD^1^

*Authors contributed equally to this work

^1^Department of Dermatology, Oregon Health and Science University, Portland OR, USA

^2^The Polyclinic, Seattle WA, USA

^3^Biostatistics Shared Resource, Knight Cancer Institute, Oregon Health and Science University

**Supplementary table 1.** Missingness among variables (columns) in the data set.

**Supplementary table 2.** Missingness among observations (rows) in the data set.

**Supplementary table 3.** General domains, linear regression results with multiple imputation.

**Supplementary table 4.** Cancer specific domains, linear regression results with multiple imputation.

This supplemental material has been provided by the authors to give readers additional information about their work.

**Supplementary table 1.** Missingness among variables (columns) in the data set

Variables (columns) in the data set that have missing data with the number of rows missing data and the percent of rows missing data.

| **Variable/column name** | **Description** | **Number missing** | **Percent missing** |
| --- | --- | --- | --- |
| ms_other_cancer | Have you ever been diagnosed with a different cancer? | 276 | 40.8% |
| obs_educ_level | What is your highest level of education? | 221 | 32.6% |
| priority_vis | Visibility of location | 213 | 31.5% |
| number_of_locations | Number of locations | 213 | 31.5% |
| melanoma_relatives | Do you have any blood relatives (not in-laws or by marriage) who have had melanoma? | 153 | 22.6% |
| age_at_qol2 | Age at survey | 100 | 14.8% |
| yrs_since_diag_f | Years since diagnosis | 100 | 14.8% |
| gender | Gender | 33 | 4.9% |
| race_f | Race | 33 | 4.9% |
| ethnic_grp | Ethnicity | 33 | 4.9% |
| ruca_scheme | Rural-Urban Commuting Area Codes | 11 | 1.6% |
| general_domains | Quality of life composite score, General Domains | 6 | 0.9% |
| cancer_specific_domains | Quality of life composite score, Cancer Specific Domains | 5 | 0.7% |
| personal_mel_stage | To the best of my knowledge, my melanoma stage was | 1 | 0.1% |

**Supplementary table 2.** Missingness among observations (rows) in the data set.

Of the 677 observations, 243 (35.9%) are completely observed and 434 (64.1%) are missing at least one variable. 22.0% are missing only one variable. 29.2% are missing 4 or more variables. One observation is missing 10 variables.

| **Number complete** | **Percent complete** | **Number missing** | **Percent missing** |
| --- | --- | --- | --- |
| 243 | 35.9% | 434 | 64.1% |

**Supplementary table 3.** General domains, linear regression results with multiple imputation.

Complete case analysis used 520 observations whereas the multiple imputation analysis retained all observations (n = 677`). The complete case model and the model from multiple imputation differ in the variables that were selected, though Stage appears in both models.

The complete case model includes Family history of melanoma, whereas the multiple imputation model does not.

For the variables that appear in both models, the estimates, confidence intervals, and statistical significance are all very similar. Either model would be suitable in our opinion. We choose to present the complete case model in the final manuscript, but limit the interpretation and the significance placed on education in that model.

|  |  | **Complete case** | | | | **Multiple imputation (65 imputed data sets)** | | | |
| --- | --- | --- | --- | --- | --- | --- | --- | --- | --- |
|  |  | **Unadjusted** | | **Adjusted** | | **Unadjusted** | | **Adjusted** | |
|  |  | **Est. (95% CI)** | **P** | **Est. (95% CI)** | **P** | **Est. (95% CI)** | **P** | **Est. (95% CI)** | **P** |
|  |  |  |  |  |  |  |  |  |  |
| Family history of melanoma |  |  | 0.033 |  | 0.017 |  |  |  |  |
|  | No | Reference level |  | Reference level |  |  |  |  |  |
|  | Yes | 4.6 (0.4 to 8.8) | 0.033 | 5.1 (0.9 to 9.2) | 0.017 |  |  |  |  |
|  |  |  |  |  |  |  |  |  |  |
| Stage |  |  | 0.006 |  | 0.001 |  | 0.008 |  | 0.008 |
|  | Stage 0 | Reference level |  | Reference level |  |  |  |  |  |
|  | Stage I+II | 3.2 (-0.6 to 6.9) | 0.095 | 3.5 (-0.7 to 7.8) | 0.103 | 3.3 (-0.5 to 7.0) | 0.088 | 3.3 (-0.5 to 7.0) | 0.088 |
|  | Stage III+IV | 11.6 (4.2 to 19.0) | 0.002 | 14.6 (6.7 to 22.5) | < 0.001 | 11.2 ( 3.8 to 18.6) | 0.003 | 11.2 ( 3.8 to 18.6) | 0.003 |

**Supplementary table 4.** Cancer specific domains, linear regression results with multiple imputation.

Complete case analysis used 402 observations whereas the multiple imputation analysis retained all observations (n = 677). The complete case model and the model from multiple imputation differ in the variables that were, though there are many variables selected by both approaches.

All variables selected for the complete case model appear in the multiple imputation model, except for RUCA designation. Gender was not in the complete case model but is in the multiple imputation model.

For the variables that appear in both models, the estimates, confidence intervals, and statistical significance are all very similar. Either model would be suitable in our opinion. We choose to present the complete case model in the final manuscript, but limit the interpretation and the significance placed on education in that model.

|  |  | **Complete case** | | | | **Multiple imputation (65 imputed data sets)** | | | |
| --- | --- | --- | --- | --- | --- | --- | --- | --- | --- |
|  |  | **Unadjusted** | | **Adjusted** | | **Unadjusted** | | **Adjusted** | |
|  |  | **Est. (95% CI)** | **P** | **Est. (95% CI)** | **P** | **Est. (95% CI)** | **P** | **Est. (95% CI)** | **P** |
|  |  |  |  |  |  |  |  |  |  |
| Age |  |  | < 0.001 |  | < 0.001 |  | < 0.001 |  | < 0.001 |
|  | 20 to 29 | 7.9 (-0.9 to 16.7) | 0.081 | 3.8 (-7.2 to 14.8) | 0.496 | 7.4 ( -1.5 to 16.2) | 0.105 | 4.0 ( -4.3 to 12.3) | 0.341 |
|  | 30 to 39 | 1.0 (-3.7 to 5.7) | 0.686 | 2.7 (-2.8 to 8.2) | 0.34 | 0.7 ( -4.1 to 5.5) | 0.783 | 0.9 ( -3.7 to 5.6) | 0.697 |
|  | 40 to 49 | 6.5 (2.5 to 10.6) | 0.002 | 7.0 (2.3 to 11.7) | 0.004 | 6.5 ( 2.4 to 10.6) | 0.002 | 6.3 ( 2.5 to 10.2) | 0.001 |
|  | 50 to 59 | Reference level |  | Reference level |  | Reference level |  | Reference level |  |
|  | 60 to 69 | -6.1 (-9.5 to -2.8) | < 0.001 | -5.8 (-9.6 to -1.9) | 0.003 | -6.1 ( -9.4 to -2.9) | < 0.001 | -4.8 ( -7.9 to -1.6) | 0.003 |
|  | 70 or older | -10.0 (-13.7 to -6.2) | < 0.001 | -8.9 (-13.3 to -4.5) | < 0.001 | -10.0 (-13.5 to -6.4) | < 0.001 | -7.5 (-11.0 to -3.9) | < 0.001 |
|  |  |  |  |  |  |  |  |  |  |
| Highest level of education |  |  | < 0.001 |  | 0.02 |  | < 0.001 |  | 0.016 |
|  | High school, equivalent, vocational training, 2-year degree | 3.7 (-0.8 to 8.2) | 0.107 | -0.2 (-4.7 to 4.3) | 0.926 | 3.9 ( 0.3 to 7.4) | 0.036 | 1.8 ( -1.6 to 5.2) | 0.307 |
|  | Some college but no degree | 1.2 (-3.2 to 5.6) | 0.597 | 0.7 (-3.7 to 5.2) | 0.751 | -1.1 (-5.0 to 2.9) | 0.597 | -0.5 ( -4.2 to 3.2) | 0.798 |
|  | A 4-year college degree | Reference level |  | Reference level |  | Reference level |  | Reference level |  |
|  | Post-graduate or professional degree | -4.3 (-7.7 to -0.9) | 0.012 | -4.5 (-7.9 to -1.2) | 0.009 | -4.2 (-7.6 to -0.8) | 0.016 | -3.6 ( -6.7 to -0.4) | 0.026 |
|  |  |  |  |  |  |  |  |  |  |
| Gender |  |  |  |  |  |  | < 0.001 |  | < 0.001 |
|  | Male |  |  |  |  | Reference level |  | Reference level |  |
|  | Female |  |  |  |  | 7.9 ( 5.4 to 10.4) | < 0.001 | 5.0 ( 2.6 to 7.4) | < 0.001 |
|  |  |  |  |  |  |  |  |  |  |
| RUCA designation |  |  | 0.085 |  | 0.02 |  |  |  |  |
|  | Urban core | Reference level |  | Reference level |  |  |  |  |  |
|  | Suburban | 1.9 (-2.4 to 6.1) | 0.388 | 1.1 (-4.3 to 6.4) | 0.691 |  |  |  |  |
|  | Large town | 3.1 (-0.8 to 7.1) | 0.116 | 4.6 (0.0 to 9.1) | 0.049 |  |  |  |  |
|  | Small town/rural areas | 7.0 (0.3 to 13.7) | 0.041 | 9.2 (2.3 to 16.2) | 0.01 |  |  |  |  |
|  |  |  |  |  |  |  |  |  |  |
| Stage |  |  | < 0.001 |  | < 0.001 |  | < 0.001 |  | < 0.001 |
|  | Stage 0 | Reference level |  | Reference level |  | Reference level |  | Reference level |  |
|  | Stage I+II | 6.3 (3.9 to 8.7) | < 0.001 | 3.4 (0.5 to 6.4) | 0.022 | 6.3 ( 3.9 to 8.7) | < 0.001 | 5.2 ( 3.0 to 7.5) | < 0.001 |
|  | Stage III+IV | 13.8 (9.1 to 18.5) | < 0.001 | 11.6 (6.1 to 17.2) | < 0.001 | 13.8 ( 9.1 to 18.5) | < 0.001 | 13.3 ( 8.9 to 17.7) | < 0.001 |
|  |  |  |  |  |  |  |  |  |  |
| Number of locations |  |  | 0.055 |  | 0.01 |  | 0.322 |  | 0.04 |
|  | 1 | Reference level |  | Reference level |  | Reference level |  | Reference level |  |
|  | 2 | 4.0 (0.4 to 7.6) | 0.031 | 5.5 (1.9 to 9.1) | 0.003 | 3.0 (-0.7 to 6.7) | 0.108 | 4.6 ( 1.3 to 7.9) | 0.006 |
|  | 3 or more | 3.6 (-1.6 to 8.8) | 0.174 | 2.6 (-2.6 to 7.8) | 0.334 | -0.3 (-4.3 to 3.7) | 0.878 | -0.5 ( -4.2 to 3.2) | 0.798 |
